# Case Report: Brain Metastasis Confined to the Infarcted Area Following Stroke

**DOI:** 10.3389/fneur.2020.617142

**Published:** 2021-01-29

**Authors:** Dong-Seok Gwak, Yang-Ha Hwang, Yong-Won Kim

**Affiliations:** ^1^Department of Neurology, School of Medicine, Kyungpook National University, Daegu, South Korea; ^2^Department of Neurology, Kyungpook National University Hospital, Daegu, South Korea

**Keywords:** metastasis, cerebral infarction, magnetic resonance imaging, renal cell carcinoma, neoplasm

## Abstract

**Background:** Ischemic stroke and cancer are frequent in the elderly and are the two common causes of death and disability. They are related to each other, and cancer may lead to ischemic stroke and vice versa. If patients with cancer exhibited recurrent acute neurological deficits after index stroke, a cancer-related stroke could be considered. However, a brain metastasis is another common cause of neurological complications and has a poor prognosis in patients with ischemic stroke and comorbid cancer. Here, we report a rare case of metastatic cancer that occurred after index stroke in a patient with renal cell carcinoma (RCC) and unusual imaging findings. Through the case, we discuss the pathophysiology and probable predisposing factors for metastatic disease in areas of infarction.

**Case Presentation:** A 48-year-old man presented with sudden onset of left facial palsy and hemiparesis. He had a history of hypertension and RCC with pulmonary metastases treated with radical nephrectomy and chemotherapy. Brain magnetic resonance imaging (MRI) revealed multiple scattered acute infarctions in the right insular, frontal, parietal, and left occipital cortices. There were no definite sources of embolism. Eight months after the index stroke, he presented with subacute onset of progressive left hemiparesis. He had no focal neurological deficits except left-sided weakness and left nasolabial fold blunting. MRI scan demonstrated partial diffusion restriction on the right frontotemporal cortices without decline of apparent diffusion coefficient values on the corresponding lesions and T1 hypointensities and T2 hyperintensities with perilesional vasogenic edema on the right insular, frontal, parietal, and left occipital cortices, indicative of brain metastases confined to the area of previous infarctions.

**Conclusions:** Cerebral infarctions can cause neovascularization and disruption of the blood–brain barrier. Moreover, the compartmentalized cavity formed by the ischemic injury may accept a large volume of metastatic tumor cells. Such an altered microenvironment of infarcted tissue would be suitable for the colonization and proliferation of metastatic seed. Further, brain metastases should be considered, in addition to recurrence, when new focal neurological deficits develop in patients with ischemic stroke and comorbid cancer.

## Background

Ischemic stroke and cancer are frequent in the elderly and are two of the common causes of death and disability (1, 2). They may occur in a patient separately, or cancer may lead to ischemic stroke and vice versa. In patients with systemic cancer, cerebrovascular complications are common. An autopsy study reported that 14.6% of patients with cancer had evidence of cerebrovascular disease (3). Moreover, the risk of ischemic stroke is elevated during the first few months after cancer is diagnosed (4). Of the patients with ischemic stroke, 3–5% develop new cancer in the 2 years after the index stroke (5–7).

The relationship between cancer and stroke is highly complex. These conditions share risk factors such as age, smoking, and obesity. Cancer may aggravate the causes of stroke, including atherosclerosis, small vessel disease, and hypercoagulability (8). Cancer treatment, including radiation therapy and chemotherapy, has been associated with increased risk of ischemic stroke (9). Furthermore, an altered microenvironment by ischemic stroke including neovascularization and blood–brain barrier leakage may be an independent risk factor for metastatic tumors (10).

Although for the patients with ischemic stroke and comorbid cancer, recent studies focused on the pathomechanisms and treatments for cancer-related stroke (8, 9, 11, 12), we should not ignore the distant metastases, including brain metastases, since their incidences may increase with the prolonged life expectancy due to improvements in cancer treatment. Further, metastases are associated with poor prognosis (13) although their underlying mechanisms have not been well-studied in this population.

Here, we report a case of metastatic cancer confined to the area of a previous cerebral infarction in a patient with renal cell carcinoma (RCC). Through this case, we discuss the pathophysiology and probable predisposing factors of brain metastasis in a patient with ischemic stroke and comorbid cancer.

## Case Presentation

**Figure 1:**
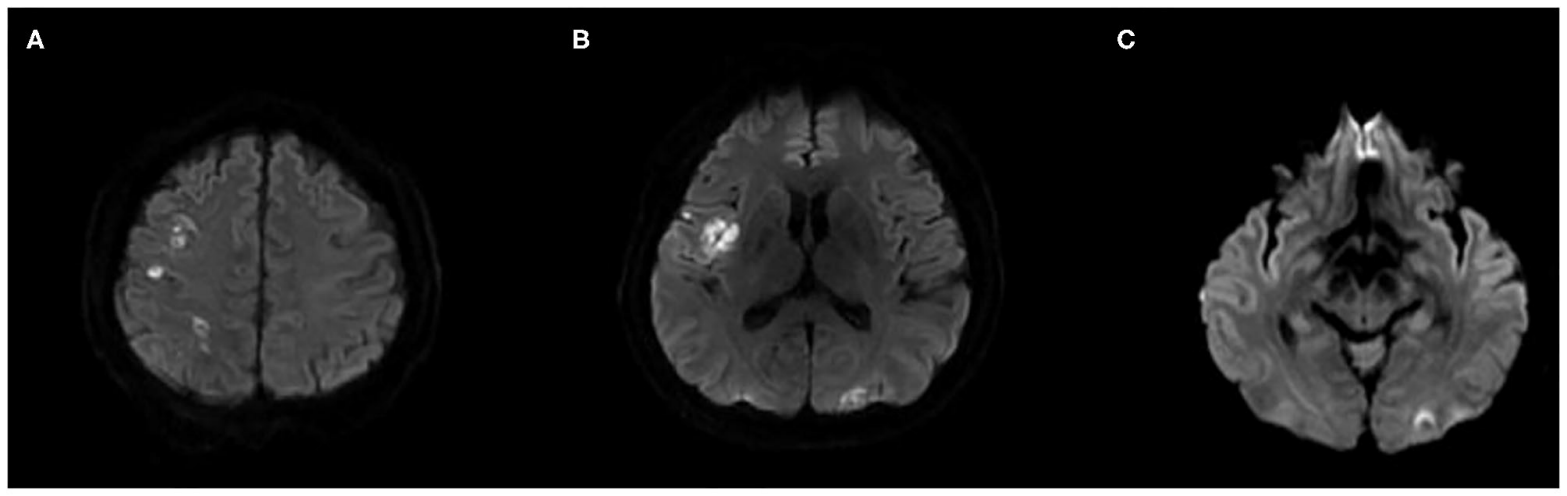
Initial brain MRI. Diffusion-weighted imaging shows multiple scattered high signal intensity on the right insular, frontal, parietal, and left occipital cortices, suggestive of acute ischemic stroke **(A–C)**.

A 48-year-old man was admitted to our hospital and presented with sudden-onset left facial palsy and hemiparesis. He had a medical history of hypertension but had no diabetes mellitus, dyslipidemia, or coronary heart disease. He suffered from pathological stage IV (T1bN0M1) RCC with pulmonary metastases. The histopathological diagnosis was clear-cell type carcinoma with grade 4 Fuhrman nuclear grading. He had been treated with radical nephrectomy and chemotherapy. Neurological examinations were otherwise normal except for left facial palsy and mild weakness in the left arm and leg [Medical Research Council (MRC) grade IV]. The baseline National Institutes of Health Stroke Scale (NIHSS) score was 3. The patient's blood pressure was 201/134 mmHg, heart rate was 94 beats per minute, and the blood glucose level was 115 mg/dL. The laboratory work included hemoglobin of 10.7 g/dL and serum creatinine of 1.25 mg/dL, but the remaining lab values were normal, including his prothrombin time (11.4 s), and partial thromboplastin time (23.9 s). Brain magnetic resonance imaging (MRI) revealed multiple scattered acute infarctions in the right insular, frontal, parietal, and left occipital cortices (Figure 1 and Supplementary Figure 1). The magnetic resonance angiography and neck computed tomography angiography study results were unremarkable. He received intravenous tissue plasminogen activator at a dose of 0.9 mg/kg. Further work-ups for evaluating stroke mechanism were done, and there was no major-risk source of cardioembolism in the cardiac evaluation, including transthoracic and transesophageal echocardiography and 24-h Holter monitoring. The presumed stroke mechanism was cancer-related hypercoagulability, and he was anticoagulated with warfarin for secondary prevention of recurrent ischemic stroke and maintained the target international standardized ratio (INR) range of 2–3. His symptoms were markedly improved during hospitalization, and the discharge NIHSS score was 0.

**Figure 2:**
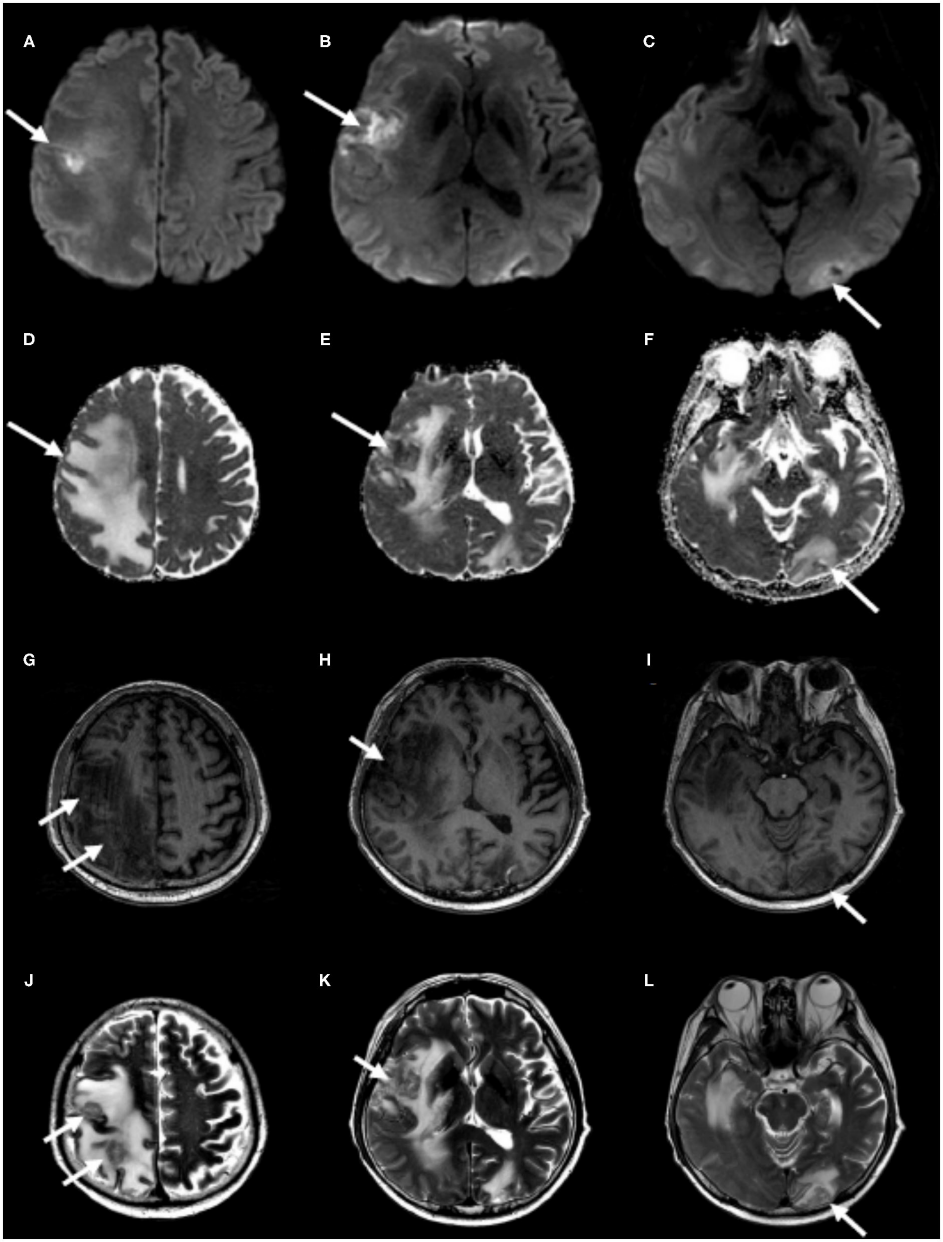
Follow-up brain MRI. Partial diffusion restriction on the right frontotemporal and the left occipital cortices **(A–C)** without decline of apparent diffusion coefficient values on the corresponding lesions **(D–F)** and hypointensities on T1-weighted images **(G–I)** and hyperintensities on T2-weighted images **(J–L)**, correlating with previously infarcted tissue (white arrows) with perilesional edema, which represents brain metastasis.

Eight months after the index stroke, he presented with left hemiparesis. His symptom progressed over 3 days before admission. He denied any other neurological symptoms, including headache, nausea or vomiting, and visual disturbances. On examination, he was alert, had no difficulties with speech, but showed a left nasolabial fold blunting and left-sided weakness (MRC grade I). His blood pressure was 139/97 mmHg, and heart rate was regular at 101 beats per minute. Laboratory findings were normal except for an INR of 3.07, hemoglobin of 8.4 g/dL, and serum creatinine of 1.98 mg/dL. The MRI scan demonstrated partial diffusion restriction on right frontotemporal and left occipital cortices without decline of apparent diffusion coefficient values on the corresponding lesions and T1 hypointensities and T2 hyperintensities with perilesional vasogenic edema on the right insular, frontal, parietal, and left occipital cortices, indicative of brain metastasis confined to the area of previous infarctions (Figure 2). He refused additional radiation therapy to the brain, received hospice care, and died within 6 months of discharge.

## Discussion and Conclusions

Brain metastases localized to infarcted tissue are rare. One study reported these metastases in a patient with uterine cervical cancer (14). To the best of our knowledge, this is the first study that describes the phenomenon in a patient with RCC. Those with advanced RCC and brain metastases generally demonstrate a poor prognosis, with a median overall survival of <1 year (15). Thus, it is important to unravel the underlying mechanisms of distant metastases to find novel strategies for preventing them and for improving clinical outcomes.

The spread of cancer cells is governed by a complex interaction between the tumor cells (seed) and the microenvironment of host organs (soil). This is the “seed and soil hypothesis” (16). Although a large number of studies have focused on the seed factors that play a role in promoting metastases *via* tumor progression and outgrowth, recent research has shed more light on the metastatic niche, which is conceptualized as a fertile soil suitable for the colonization and proliferation of metastatic seed. The common functions of the metastatic niche include anchorage, pro-survival signaling support, protection from external insults, and fostering proliferation (17).

Cerebral infarction can cause neovascularization and disruption of the blood–brain barrier (10, 14). The infarcted tissues featured copious small capillary-sized vessels lined by plump endothelial cells (14). One experimental study suggested associations between neovascularization and hyperemia (18). These properties could lead circulating tumor cells to selectively lodge in the newly formed vascular network of infarcted regions and easily penetrate the brain parenchyma. Furthermore, the compartmentalized cavity formed by the ischemic injury may accept a large volume of metastatic tumor cells. Such an altered microenvironment of infarcted tissue would be compatible with the characteristics of fertile soil and have the potential to be a metastatic niche.

This study has several limitations. It is based on a single case report, and we did not perform histopathological confirmation tests of the brain metastases. Moreover, due to worsening of renal function of the patient at readmission, gadolinium-enhanced MRI could not be done, which made it difficult to interpret MRI findings. Despite these limitations, our finding of metastatic tumors confined to the regions of previous cerebral infarction support the seed and soil hypothesis and the hypothesis that infarcted tissue can be a metastatic niche. Further, brain metastasis should be considered, in addition to recurrence, when new focal neurological deficits develop in patients with ischemic stroke and comorbid cancer.

## Data Availability Statement

The datasets used and analyzed during the present study are available from the corresponding author on reasonable request.

## Ethics Statement

Ethical review and approval was not required for the study on human participants in accordance with the local legislation and institutional requirements. The patient next of kin provided written informed consent to participate in this study.

## Author Contributions

D-SG, the first author, interpreted the data and wrote the manuscript. Y-HH revised the manuscript for intellectual content. Y-WK designed and conceptualized study, interpreted the data, and drafted the manuscript for intellectual content. All authors have read and approved the manuscript.

## Conflict of Interest

The authors declare that the research was conducted in the absence of any commercial or financial relationships that could be construed as a potential conflict of interest.

## Supplementary Material

Supplementary Figure 1 Initial brain MRI. Apparent diffusion coefficient shows low values on the right insular, frontal, parietal, and left occipital cortices **(A-C)**, iso- to hypointensities on T1-weighted images **(D-F)** and hyperintensities on T2-weighted images on the corresponding lesions **(G-I)**. Together with high signal intensity in diffusion-weighted imaging, these findings are suggestive of acute cerebral infarction.
