# Supplementary figures and images for: Case Report: Brain Metastasis Confined to the Infarcted Area Following Stroke
Source: Front Neurol. 2021 Jan 29;11:617142. doi: 10.3389/fneur.2020.617142 (PMC7878549; doi:10.3389/fneur.2020.617142)

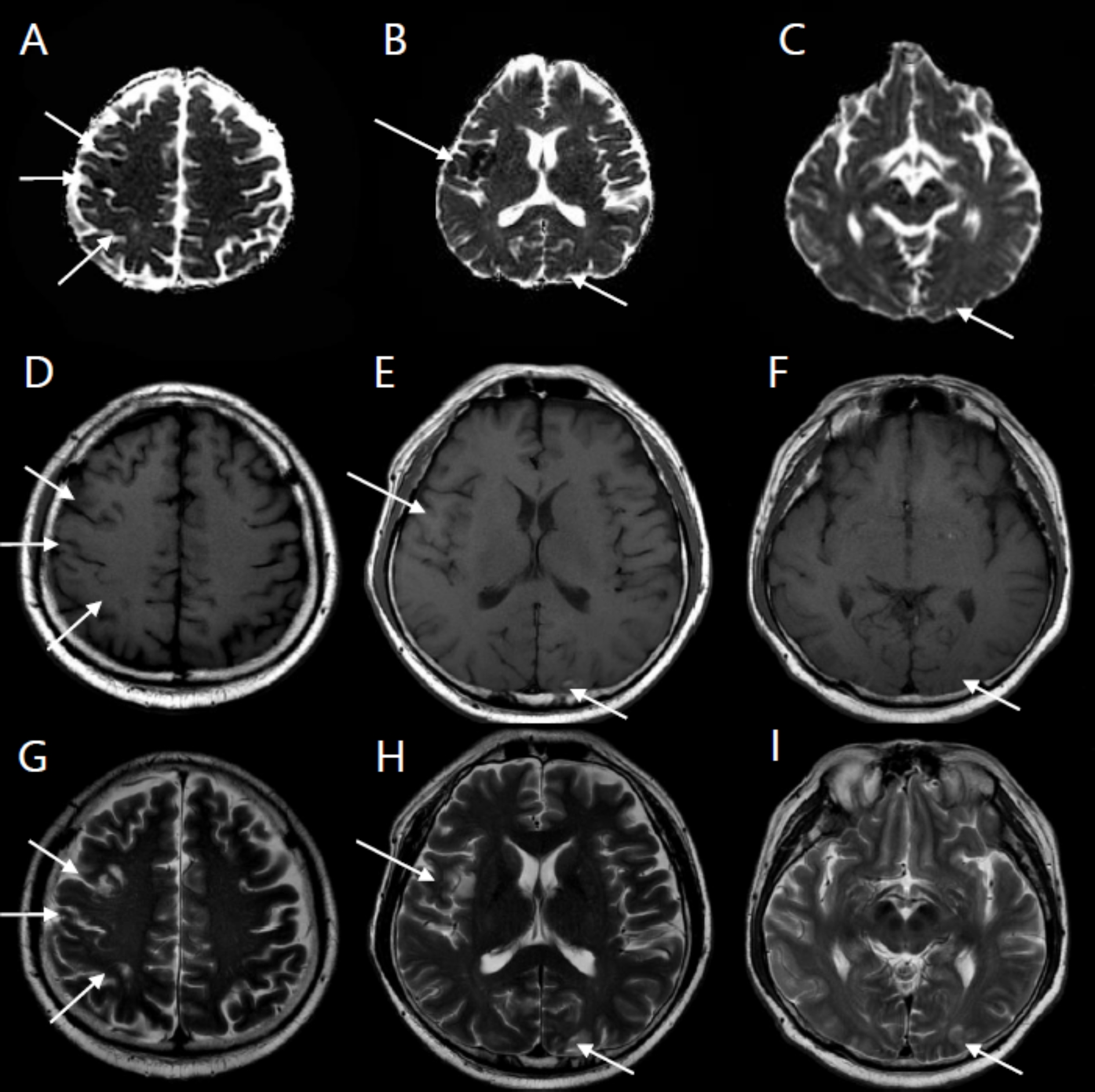

Supplement: Supplementary Figure 1 — Initial brain MRI. Apparent diffusion coefficient shows low values on the right insular, frontal, parietal, and left occipital cortices (A-C), iso- to hypointensities on T1-weighted images (D-F) and hyperintensities on T2-weighted images on the corresponding lesions (G-I). Together with high signal intensity in diffusion-weighted imaging, these findings are suggestive of acute cerebral infarction. [file Image_1.JPEG]
